# Dry eye disease immune responses and topical therapy

**DOI:** 10.1186/s40662-019-0137-2

**Published:** 2019-04-22

**Authors:** Charles W. McMonnies

**Affiliations:** 1 School of Optometry and Vision Science, University of New South Wales, Kensington Sydney, 2052 Australia; 2 School of Optometry and Vision Science, 77 Cliff Avenue, Northbridge Sydney, 2063 Australia

**Keywords:** Dry eye disease, Topical drugs, Cyclosporine

## Abstract

There is accumulating evidence that inflammation is one of the key components of dry eye because chronic ocular surface inflammation can be both a result as well as an initiator of dry eye. The need for continuing anti-inflammatory therapy may be determined in part by the extent that non-modifiable factors such as gender and age-related aqueous or lipid or mucus production deficiencies contribute to its chronicity. This perspective examines how the need for increased dosage of a topical anti-inflammatory drug may be determined by the degree of difficulty that a topically administered drug has in accessing different sites of tear deficiency and associated inflammation.

## Background

Risk factors for dry eye disease (DED) can be classified as modifiable and non-modifiable [1]. Modifiable factors can include adverse environmental conditions such as exposure to air-conditioning and those which cannot be controlled include sex- and age-related reduced aqueous tear and lipid production and as well as the influence of climatic conditions. The need for continuing DED therapy may be determined by the extent of non-modifiable factors contributing to its chronicity. Inflammation is one of the key components of DED [2] although chronic ocular surface inflammation can be a consequence as well as a propagator of DED [3]. Epithelial cells play a key role in the persistence and even the initiation of chronic ocular surface inflammation [4]. Immune responses and inflammation initiated by ocular surface desiccating stress for example, can generate immune response cascades which lead to additional surface damage and a self-perpetuating inflammatory cycle [3] that may amplify toward the end of each day [5]. Ideally, breaking or slowing the inflammatory cycle can be achieved by addressing initiating mechanisms such as tear hyperosmolarity due to inflammation-related lacrimal gland (LG) and/or meibomian gland (MG) dysfunctions and/or mucous deficiencies.

## Main text

Cyclosporine A (CsA) is an immunomodulatory drug [3] which has been found to mediate immunosuppressive effects primarily through the immune cells [6]. CsA can be used to reduce ocular surface immune responses and inflammation [7]. However, as examined below, a topical treatment may have delayed and/or less influence on LG and MG dysfunction which can initiate the development of an amplifying inflammatory hyperosmolarity-based dry eye cycle [5]. Ideally, treatment for DED is targeted at the specific mechanisms which are driving the disease mechanisms in each patient [8]. This review includes an examination of the potential for CsA, as an exemplar topical drug for DED, to reduce inflammation associated with tear hyperosmolarity resulting from LG deficiency (LGD), MG deficiency (MGD) and/or mucous deficiencies.

Topical administration of ocular therapy is expected to avoid the side effects associated with systemic delivery of drugs such as CsA [9]. Reviews have found that most studies have demonstrated improvement in at least some symptoms of dry eye in patients treated with topical CsA [10, 11]. Following topical administration, CsA is expected to achieve effective drug concentrations in the immune response and inflammatory target areas [12]. However, access and penetration appears to be easiest for the ocular surface followed by the MGs and then the LGs, although rapid clearance from the tears presents a formidable obstacle to the delivery of drugs to even the ocular surface [13]. All of the potential barriers to drug penetration from topical administration had a total drug loss of about 95% with the remainder encountering corneal and conjunctival epithelial barriers [14]. More frequent dosing may be used to improve bioavailability of topical drugs [10] for sites which are more difficult to access. For example, CsA four times a day has been used to treat refractory cases of blepharoconjunctivitis in children [15]. Moderate to severe dry eye was treated with topical CsA twice daily for six months with a significant reduction in the number of activated lymphocytes within the conjunctiva (*p* < 0.05) [7]. Frequency of dosing may be inversely related to duration of treatment and the potential for commencing tapered dosage.

Favourable corneal and conjunctival responses to CsA appear unlikely to represent similar reductions in inflammatory activity in LGs or MGs which are not as easily accessed as the ocular surface by topical therapy. Access to the MGs is more difficult because the conjunctival epithelium acts as a significant barrier and, as discussed below, there appears to be even greater difficulty of access to the LG. Reduced levels of access to these sites of inflammation may be the basis for long periods of therapy used in CsA trials with beneficial effects sometimes not being demonstrated until at least the two-month visit in studies of MGD for example [16]. Again, increased frequency of dosing may shorten response times at more difficult to access sites of DED pathology.

Early trials with a series of topical CsA formulations which were intended to improve penetration, were found to be inefficient in reaching intraocular targets [9]. More recently, Schopf and co-authors showed how mucus-penetrating particles could be used to enhance exposure of ocular tissues and the aqueous humor to a topically applied corticosteroid [17]. Nevertheless, drug access to an extra-ocular target such as the LG may be even more problematic. Fine excretory ducts pass from both portions of the LG to open by ten to twelve small orifices just in front of the outer part of the superior fornix with one or two also opening into the outer part of the lower fornix [18]. For topically delivered CsA, the direction of flow of new tears onto the ocular surface through the LG ducts appears likely to limit access for CsA to the palpebral portion but especially the orbital portion of the LG. However, as much as 30% of the volume of LG fluid can be secreted by the duct cells [18] which have more favourable access to topical CsA. For patients with aqueous deficiency, long latent periods before patient reported improvements in symptoms [10] may be explained by limited access of the drug to the LG. That permeability of the conjunctiva can be greater than that of the cornea [19] suggests that a passage for CsA through the fornical conjunctiva to the LG is feasible in a manner that is somewhat similar to the delivery of CsA through the palpebral conjunctiva to the MGs.

In patients for whom immune responses and inflammation are core mechanisms of their DED, the key to successful CsA treatment may be to prescribe dosing according to the degree of access to the prime source or sources of inflammation. Reduced inflammation in the more easily accessed cornea and conjunctiva may more easily be associated with symptomatic relief. However, such improvement may be more likely to dissipate when treatment ceases depending on the degree that LGs and MGs are dysfunctional and on the extent that associated inflammation in those parts has not yet been sufficiently reduced. If the prime source of inflammatory activity is the LGs and/or the MGs, and ocular surface inflammation is a consequence of hyperosmolarity associated with such inflammation, then the benefit of reducing ocular surface inflammation may rapidly dissipate when immunomodulatory therapy ceases because tear osmolarity may remain at pathological levels. The rate of dissipation of immunomodulatory benefits following cessation of CsA treatment appears to be unknown. The DEWS II review found that CsA treatment needs to be continued for extended periods, as evidenced by the rarity of an absence of symptoms following drug discontinuation [3]. It is possible that patients can become dependent on immunomodulatory treatment. However, the same consideration applies to other forms of treatment for DED such as lubricant therapy. Also, MGD treatment to reduce the influence of lipid deficiencies likely needs permanent maintenance to avoid relapse to lipid deficiency.

Utine and co-authors reported no serious side effects associated with topical CsA treatment up to twelve months [20]. However, the review of Wan and co-authors found that more adverse effects occurred in patients treated with topical CsA compared with controls [11]. The most commonly reported side effect associated with CsA has been ocular burning on administration [10] which may be classed as non-serious when balanced against the level of any favourable long-term responses. However, ocular burning on instillation can be a serious side effect if it results in reduced compliance with recommended dosing frequency or even the abandonment of therapy. Also, inflammatory responses to burning on instillation make the anti-inflammatory task for a drug such as CsA that much harder. Reflex tearing would more rapidly dilute drug concentration. Any instillation discomfort may be a stronger influence on discontinuation of therapy when patients move from a free supply of CsA during a trial therapy period to a post-trial financial burden. Cost considerations depend on the degree of satisfaction with responses to other forms of treatment that may be expensive to maintain. For example, CsA treatment may provide some cost advantage for DED patients who are unresponsive to lubricant therapy [21].

## Discussion

Due to the influence of factors such as sex, age and genetics, there is notable interindividual heterogeneity in drug responses which determines both drug efficacy and toxicity [22]. Such variables might help explain indeterminate findings in some studies of CsA treatment for DED. In addition, heterogeneity of responses may also be due to an inappropriate standard dosing of topical CsA for all moderate to severe subjects irrespective of the type of DED. As mentioned above, treatment is best targeted at the specific mechanisms which are driving the DED process in each patient [8]. For example, tear instability due to goblet cell dysfunction-related mucin deficiency is directly related to chronic inflammation and surface cell apoptosis that is subsequent to cell hyperosmolarity [23]. Reducing more easily accessed ocular surface inflammation may require less frequent dosing of topical CsA to achieve improved secretory and/or trans-membrane mucus production. However, to the extent that ocular surface inflammation is a consequence of hyperosmolarity due to LG and/or MG dysfunction, breaking an amplifying DED inflammatory cycle [5] would appear to require treatment which adequately addresses those dysfunctions appropriately. Rapid responses to topical anti-inflammatory therapy [24] may be the outcome of an initial ocular surface response due to easier access to corneal and conjunctival inflammation. However, these responses may not be sustained until any more difficult to access LG or MG inflammation has also been reduced. A review of the possibility of significant levels of topically administered CsA being found in blood reported that it was detected in the blood samples of only 2% of subjects using a 0.1% dose of topical CsA and in none of the subjects using a 0.05% dose [25].

## Conclusions

This perspective may have relevance to topical anti-inflammatory/immunomodulatory drugs other than CsA which are used to treat DED. Based on a clinician’s success in identifying the types of tear deficiency and the sites of inflammatory activity which are relevant for a patient, the dosage for an anti-inflammatory drug which is administered topically may be usefully varied according to the accessibility to those sites of inflammation. For example, rather than placing too much dependence on a reduction in ocular surface immune responses, which are downstream in an amplifying hyperosmolarity-based DED cycle, increased dosing frequency with topical CsA might be necessary for evaporative DED involving MG inflammation, but more so in the case of aqueous deficient DED involving the seemingly even more difficult to access inflammation in the LG. Tapered dosing of topical anti-inflammatory drugs may be appropriate for preventing relapses in cases which involve non-modifiable causal factors.
